# “It’s a black box”: Physiotherapists’ perspectives on sensorimotor control and the use of technology in this context to improve rehabilitation following ACL injury

**DOI:** 10.1186/s12891-026-10484-x

**Published:** 2026-09-23

**Authors:** Sanna Mikko, Anna Cronström, Marlene Sandlund, Charlotte K. Häger

**Affiliations:** 1 Department of Community Medicine and Rehabilitation, Umeå University, Umeå, Sweden; 2 Department of Health Sciences, Lund University, Lund, Sweden

**Keywords:** ACL, Movement quality, Movement control, Wearable technology, Portable technology, Digital technology, Physiotherapy, Qualitative study, Focus group

## Abstract

**Background:**

Anterior cruciate ligament (ACL) injuries commonly occur in non-contact situations, implicating that underlying deficits in sensorimotor control may contribute to both primary and secondary injury risk. Physiotherapists play a central role in rehabilitation and have opportunities to advance recovery by use of technology targeting sensorimotor control. The integration of technologies such as wearable sensors, virtual reality, and artificial intelligence methods may support more objective assessment and context-related training after ACL injury, but clinical implementation remains limited. This study explored physiotherapists’ perspectives on sensorimotor control, including their view on how technology may support its assessment and rehabilitation following ACL injury.

**Methods:**

A qualitative design was used, comprising six digital focus group discussions with 22 physiotherapists experienced in ACL rehabilitation (median experience 7 years, range 3–27), with current positions including generalist physiotherapists, clinical specialists and researchers. Discussions were recorded, transcribed, and analysed using an inductive approach and reflexive thematic analysis (Braun and Clarke).

**Results:**

The analysis generated three themes: (1) Calling for measurable indicators of movement quality (2), Integrating technology as a meaningful complement to clinical assessment, and (3) Extending rehabilitation beyond the clinical walls. The physiotherapists called for methods to assess movement quality with outcome measures relevant to functional recovery and ACL re-injury risk. Although technologies were viewed as potentially supportive for assessment and rehabilitation, their added clinical value remained somewhat unclear. Adoption of technology was perceived to depend on the demonstration of clear clinical benefits, integration with clinical reasoning, and adaptation to emerging shifts in rehabilitation structures.

**Conclusions:**

The physiotherapists expressed interest in technology to support sensorimotor assessment and rehabilitation after ACL injury. However, implementation was limited by uncertainty regarding the clinical value of sensorimotor outcomes. Future research needs to establish sensorimotor outcomes that are relevant for functional performance and/or re-injury risk and investigate whether educating physiotherapists in emerging technologies will improve the understanding of their potential and limitations as well as stimulate increased use. Co-creation between physiotherapists, researchers, and technology developers may help align emerging technologies with clinical workflows and reasoning.

**Supplementary Information:**

The online version contains supplementary material available at https://doi.org/10.1186/s12891-026-10484-x.

## Background

Rupture of the anterior cruciate ligament (ACL) is common among sport-active individuals [1]. Despite undergoing extensive physiotherapist-led rehabilitation, persistent physical and psychological barriers often limit return to pre-injury levels of sport and physical activity [2, 3]. Approximately 20% of those who return to sport (RTS) sustain a second ACL injury [4], highlighting the need for improved rehabilitation strategies. Research indicates variation in current clinical practice, and there is a lack of consensus regarding assessment and rehabilitation guidelines [5–8].

A substantial proportion, approximately 60–85%, of ACL injuries occur via non-contact or indirect contact situations such as rapid changes of direction, landing or deceleration in certain sporting contexts [9, 10], suggesting that impaired sensorimotor control plays an important role in both primary injury and re-injury risk. Sensorimotor control refers to the integration of sensory information, central nervous system processing, and motor output to plan, adjust, and refine movement in real time [11]. Following ACL injury, sensorimotor function may be disrupted due to loss of proprioceptive information from damaged mechanoreceptors of the native ACL, mechanical instability, pain, inflammation, and psychological factors such as fear or anxiety related to movement [12–14]. These disruptions may manifest as aberrant movement patterns, compensatory muscle co-contraction strategies, impaired postural control, and joint position sense [15–18], each potentially increasing re-injury risk. In clinical practice, these observable aspects are often described in the context of addressing movement quality [19]. Targeting sensorimotor recovery is therefore crucial, yet there is a lack of appropriate methods for assessing and addressing this in the clinics [20, 21].

In response to these challenges, recent technological advancements have expanded the opportunities for detailed and contextually relevant assessment of sensorimotor control in the rehabilitation of individuals following ACL injury [22]. While laboratory-based motion capture systems, including force plates and electromyography, remain the gold standard for movement analysis, their limited portability and high cost restrict clinical use. More affordable and portable solutions, such as pressure insoles and video-based motion capture systems, have therefore emerged [22, 23], increasing accessibility and facilitating their use in clinical settings. Emerging research suggests that technologies such as wearable sensors, pressure insoles, virtual reality (VR), and artificial intelligence (AI)-based tools may support assessment and rehabilitation following ACL injury by enabling movement monitoring, exposure of at-risk biomechanics, multisensory training, and individualised feedback [24–26]. Potential clinical applications include objective assessment of between-limb asymmetries, biofeedback to optimise motor learning, monitoring of recovery over time, and support for clinical decision-making throughout rehabilitation [22, 24, 27]. However, despite this potential and generally high technology acceptance among physiotherapists, implementation of technological systems in physiotherapy practice, in general, remains limited due to practical and organisational barriers [28–30]. Incorporating physiotherapists’ perspectives is therefore essential to understand how technological solutions can be made meaningful and applicable in practice and to explore how they fit with physiotherapists’ needs, priorities, and clinical reasoning [31].

Despite physiotherapists’ central role in evaluating sensorimotor control and guiding RTS decisions in ACL injury management, limited knowledge exists regarding how these evaluations are integrated into clinical practice. Moreover, although technological solutions are increasingly being developed [22, 32], it remains unclear how physiotherapists interpret and use the resulting data in their clinical reasoning.

Therefore, we aimed to explore physiotherapists’ perspectives on sensorimotor control, including their views on how technology may support its assessment and rehabilitation following ACL injury.

## Method

### Study design

A qualitative study was conducted with focus group discussions involving physiotherapists experienced in ACL rehabilitation. Focus group methodology was chosen to gather diverse viewpoints and enable exchange of ideas among the physiotherapists that could reveal additional aspects of the topic, in line with a social constructivist orientation [33]. The analytic approach followed Braun and Clarke’s reflexive thematic analysis with an inductive approach [34, 35]. The Consolidated Criteria for Reporting Qualitative Research (COREQ) guided the reporting [36].

### Recruitment and participants

Clinically active, Swedish-speaking physiotherapists who had worked with ACL rehabilitation for at least one year were eligible. The one-year criterion was chosen to ensure some experience of ACL rehabilitation while still allowing inclusion of recently graduated physiotherapists who may contribute with valuable perspectives. Recruitment was conducted through the Swedish ACL injury network, social media, the authors’ professional networks, and snowball sampling. This purposive sampling strategy was used to achieve variation in gender, age, geographic location, workplace, and research/clinical experience, with particular emphasis on orthopaedics and sports medicine specialists. In total, 133 physiotherapists were invited by email. Thirty physiotherapists expressed interest. Of these, eight did not participate due to either scheduling conflicts (*n* = 5) or personal choice (*n* = 3), resulting in 22 participants being included in the study. All participants received written study information, provided informed consent, and submitted demographic data by email prior to participation. They had 3–27 years of experience in ACL rehabilitation and worked in rehabilitation centres, orthopaedic surgery clinics, private clinics, and primary care. They cared for patients ranging from recreational to elite levels across eleven Swedish regions, from north to south (Table 1).

**Table 1:** Participant characteristics

Characteristics	Data
Number of physiotherapists	22 (12 females, 10 males)
Age, median (range)	36 (29–60)
ACL rehabilitation experience, years, median (range)	7 (3–27)
Further education	Master’s degree (*n* = 11), clinical specialist exam (*n* = 6)
Research background (minimum doctoral student)	*n* = 7
Self-reported areas of professional interest	Sports medicine (*n* = 18), knee pathologies (*n* = 8), orthopaedic manual therapy (*n* = 4), orthopaedics (*n* = 3), women’s health (*n* = 2), musculoskeletal health (*n* = 1), pain (*n* = 1)
Work setting	Rehabilitation centre (*n* = 12), orthopaedic surgery clinic (*n* = 5), private clinic (*n* = 3), primary healthcare centre (*n* = 2)

### Data Collection

Six digital focus groups held in Swedish were conducted between January 2023 and September 2024 using video and audio communication, with audio recorded using an external device. Each focus group included 2–6 physiotherapists and lasted 90–105 min. Group sizes varied due to recruitment and scheduling constraints. The first author (SM) facilitated all focus groups, and a co-facilitator (AC) assisted in the first four sessions. A semi-structured interview guide was developed by the research team specifically for this study, informed by a literature review, team discussions, and feedback from an external expert in qualitative methodology (Supplementary file 1; Fig. 1). The guide covered four main areas: physiotherapists’ perspectives of sensorimotor control, technological aids, remote rehabilitation, and future directions for technological development. Each session began with a short overview of the study aims followed by participant introductions and an opening question to encourage initial discussion. To minimise potential power imbalances, the authors clarified their role as facilitators and emphasised that the discussions were not intended to assess knowledge but an opportunity to share diverse professional perspectives, with all contributions valued. The first focus group was included in the analysis and served to pilot the interview guide. Minor refinements followed, including the addition of brief theoretical introductions on sensorimotor control and technological aids to contextualise the discussion [11, 37–39], delivered with support from a PowerPoint presentation. After focus group 4, the research team reflected on information power of the data [40]. The initial focus group discussions were intentionally broad, exploring physiotherapists experiences and perspectives on ACL rehabilitation in general. Preliminary analysis of these discussions indicated that the technology-related aspects had not been examined in sufficient depth. Consequently, two additional focus groups were conducted using a slightly revised discussion guide with stronger emphasis on experiences with and future perspectives on technology in ACL injury rehabilitation. Topics included for instance wearable sensors, VR, AI-based methods, and digital health solutions. This enabled a more focused and in-depth exploration of technology-related perspectives. Identifiable information was pseudonymised using participant codes. The facilitators had prior professional familiarity with a total of three physiotherapists included in the study.

### Data analysis

Data were analysed using reflexive thematic analysis according to Braun and Clarke with an inductive approach [34, 35]. MAXQDA 2022 (VERBI Software, Berlin, Germany) was used to support data management and coding. The analysis followed the six phases described by Braun and Clarke and included familiarisation with the transcripts, generation of semantic and latent codes, organisation of codes into candidate themes, iterative refinement through team discussion, development of final themes and sub‑themes with illustrative quotes and reporting text as presented in Table 2. Coding was conducted in Swedish by SM, reviewed by MS, and regularly discussed with the other co-authors to enhance methodological rigour. Theme names and the narrative reporting text were developed in English among all authors. Throughout the analytic process, transcripts, codes, and themes were continuously revisited to support contextual understanding and credibility of the findings [34]. Selected quotations were translated by a native English-speaking researcher with expertise in ACL injuries. Translations were cross-checked with the Swedish originals to preserve meaning and nuance. Triangulation was achieved through regular author discussions that integrated diverse forms of pre-understandings. These pre-understandings included clinical and personal experiences of ACL injury rehabilitation (SM, Doctoral Student), laboratory-based research on sensorimotor control in ACL populations and qualitative methodology (AC, Associate Professor), extensive expertise in qualitative research on digital health beyond ACL injuries (MS, Professor), and more than three decades of research on sensorimotor control and health technology combined with clinical orthopaedic practice and experience of qualitative research methods (CH, Professor). All authors were female physiotherapists. Peer debriefing was conducted with several researchers holding insider and outsider perspectives such as two doctoral students (informatics and physiotherapy) and an orthopaedic nurse with a doctoral degree. One male physiotherapist from the focus groups also performed a member check of the preliminary findings. Their feedback helped clarify theme boundaries and refine the wording of selected interpretations, while the overall structure of the findings remained unchanged.

**Table 2:** Examples of the analysis process

Raw data	Code	Sub-theme	Theme
*“First*,* we would need to map things out a bit more. For example*,* what does it mean when a knee valgus angle of 5° or 10° is observed relative to the hip joint? Does the individual generate more force that way?”* (FG4 - P11)	Need to understand movement patterns in more detail	Navigating movement quality in the absence of meaningful outcomes	Calling for measurable indicators of movement quality
*“Almost all technological aids I’ve encountered tend to overestimate the patient’s function a bit… you have to be a little more realistic.”* (FG5 - P14)	You can’t rely solely on the results from the technology	Balancing technological objectivity with professional judgement	Integrating technology as a meaningful complement to clinical assessment
“*If they [patients] were able to wear sensors*,* especially regarding the side-to-side differences… standing*,* walking*,* and everyday movements that are difficult to monitor digitally [digital patient meetings] … those kinds of sensors would have added much clearer information.”* (FG2 - P6)	Would like to complement evaluation from digital meetings with sensor information	Developing clinical solutions for monitoring and feedback	Extending rehabilitation beyond the clinical walls

*FG* refers to Focus group, *P* refers to participant

## Results

We identified three themes that outlined the physiotherapists’ perspectives on sensorimotor control and use of related technologies to improve rehabilitation following ACL injury. The themes were: (1) Calling for measurable indicators of movement quality (2), Integrating technology as a meaningful complement to clinical assessment, and (3) Extending rehabilitation beyond the clinical walls. Within these themes, seven sub-themes were generated (Table 3), illustrated with verbatim quotes below. Notably the physiotherapists mostly expressed themselves in term of movement quality in the context, which is reflected in the themes and quotes. Supplementary file 1; Table 1 provides illustrative examples of technologies discussed by participants, including their current use and envisioned applications.

**Table 3:** Themes and sub-themes with illustrative quotations of physiotherapists’ perspectives on sensorimotor control and related technology use in ACL rehabilitation

Themes	Sub-themes	Illustrative quotations
Calling for measurable indicators of movement quality	Navigating movement quality in the absence of meaningful outcomes	*“We have an idea of a good movement pattern*,* but what is a good movement pattern really?”* (FG6 - P18)
	Using technology to enhance understanding of movement quality	*“Like that force mat. There you have it in black and white… Having a few more measurement instruments would be a dream.”* (FG1 - P4)
Integrating technology as a meaningful complement to clinical assessment	Justifying the added clinical value of implementing technology	*“Force plate combined with 3D markers… That would be awesome. But then how would I actually use it [the technology]? I’m not really sure I dare to answer that.”* (FG1 - P2)
	Balancing technological objectivity with professional judgement	*“It’s important not to lose your own judgement. It [the technology] should just be seen as a complement.”* (FG6 - P22)
	Utilizing increased technology-based information about the patient	*“Testing and collecting data is one thing*,* but we also need to do something with it.”* (FG6 - P18)
Extending rehabilitation beyond the clinical walls	Mimicking sport-like demands requires adaptations in clinical practice	“*There is a huge gap between the clinic and the sports environment. That type of training could be developed further*,* so that you are more involved when they [the patients] are exposed to what they actually want to do*.” (FG4 - P11)
	Developing clinical solutions for monitoring and feedback	*“So that you can get a graph… Having some easy-to-understand data to show the patient and evaluate for the next appointment. You’ve become stronger and gained better balance*,* then this graph has also improved.”* (FG4 - P10)

*FG* refers to Focus group, *P* refers to participant

### Calling for measurable indicators of movement quality

The physiotherapists described uncertainty regarding how movement quality should be evaluated in clinical practice and discussed whether technologies such as wearable sensors and AI could support more clinically relevant assessment. Two sub-themes were generated: a) Navigating movement quality in the absence of meaningful outcomes and b) Using technology to enhance understanding of movement quality.

#### Navigating movement quality in the absence of meaningful outcomes

The physiotherapists described desirable movement quality as involving body symmetry, optimal joint alignment, coordinated muscle activation patterns, and smooth movement execution. Despite this view, they felt that the underlying evidence for what defines good movement quality was insufficient, particularly regarding how these aspects relate to improved function and reduced risk of re-injury. Since they valued evidence-based practice, this lack of evidence contributed to frustration and uncertainty about what is important to assess, and how clinical findings should be interpreted and integrated into the individual rehabilitation.

*“It’s a bit of a headache… is what we’re measuring even relevant for new injuries? It’s a black box that we can’t control.”* (Focus group 1 - participant 2).

The perceived limited evidence also seemed to underpin the varying approaches used when evaluating movement quality. Some relied on structured criteria, while others used intuitive judgement, which they described as difficult to articulate or quantify. Clinical experience was considered important, and the physiotherapists noted that approaches could vary depending on clinicians’ backgrounds and previous patient observations. Views also differed on the importance of correction of movement patterns, ranging from considering it as essential to improve key skills, to doubting its’ value due to uncertainty about linking these changes to meaningful clinical outcomes.

*”To perform a nice turn or to jump up and land in a good way*,* for me*,* that’s extremely important. I work a lot with that.”* (Focus group 6 - participant 21) /…/ *”I think there’s too much focus on balance and control if I’m being honest…if you spend a lot of time correcting that*,* you miss valuable time that could have been spent on other things.”* (Focus group 1 - participant 2).

#### Using technology to enhance understanding of movement quality

The physiotherapists reflected on how emerging technologies could support more objective assessments of movement quality, and thereby strengthening the evaluation of related training interventions while clarifying their clinical relevance. Wearable sensors, video analysis, and electromyography were discussed as tools to enable detailed biomechanical analysis during complex, sport-specific tasks beyond visual observation. The physiotherapists also described technology as a necessary means to translate findings from advanced movement laboratories into clinically feasible assessment approaches. Alongside biomechanical assessment, the physiotherapists highlighted that psychological factors may influence movement quality and emphasised digital collection of patient-reported outcome measures to support a more comprehensive evaluation. The physiotherapists also reasoned about the potential of AI to integrate patient information from multiple assessment approaches. Such analyses were viewed as having potential to identify clinically meaningful changes over time, particularly related to patient performance and risk of re-injury. At the same time, the physiotherapists highlighted that movement strategies may vary between individuals and across contexts, underscoring the importance of maintaining an individualised approach when interpreting AI-supported analyses.

*“…a development of different video tools…3D analyses…linked to force plates…to get a comprehensive biomechanical analysis…that I think would be interesting to work towards.”* (Focus group 6 - participant 19) /…/ *“To be able to record videos to assess jumping and landing ability… this type of movement analysis isn’t easy to do accurately*,* even for a trained eye*,* so perhaps AI could help. Maybe even out in the [sport] field.”* (Focus group 6 - participant 20).

### Integrating technology as a meaningful complement to clinical assessment

The physiotherapists expected technology to become more common in rehabilitation but emphasised that its usefulness depends on how well it supports clinical work. Accordingly, three sub-themes were generated: (a) Justifying the added clinical value of implementing technology, (b) Balancing technological objectivity with professional judgement, and (c) Utilizing increased technology-based information about the patient.

#### Justifying the added clinical value of implementing technology

The physiotherapists expressed uncertainty about whether available technologies offered enough clinical value to justify their adoption. Some physiotherapists were already working with tools such as force plates, the MuscleLab system or smartphone-based applications, while others described being at a crossroads, unsure what these tools added to clinical reasoning. Limited awareness of available technologies further contributed to this uncertainty.

*“I don’t know*,* really. The question is*,* what does it [technological tools] actually add? Are we evaluating that thoroughly? Does it bring any value? I don’t know.”* (Focus group 5 - participant 13).

Growing societal expectations and patients’ trust in technology were perceived to increase pressure on clinics to adopt new tools. Some physiotherapists described a need to keep pace with technological developments to appear up to date, while also perceiving clinical value in more objective assessment approaches, including the quantification of asymmetries.

*“We’ve recently acquired a force plate… you want to stay at the forefront… we use hop tests*,* but we know that many patients can perform well on them using different*,* less optimal movement strategies.”* (Focus group 1 - participant 3).

At the same time, the physiotherapists emphasised that bringing new technological tools into practice required financial investment, time and effort. Such tools had to be updated, set up and taken down between patients, making it essential to justify their added value. To support meaningful implementation, they highlighted the need for technological tools that are based on evidence.

#### Balancing technological objectivity with professional judgement

The physiotherapists emphasised that technological data must be balanced with professional judgement to retain clinical relevance. Technology was described as useful only when it complements, rather than replaces, professional judgement. Some physiotherapists expressed concern that increasing reliance on objective measures could undermine core clinical skills, particularly when technological outputs did not fully reflect patients’ functional performance. They described situations where technology appeared to overestimate functional ability, making clinical reasoning essential for interpreting results in patient contexts. Being directly involved in the assessment was viewed as crucial for detecting subtle behaviours, such as hesitation, discomfort or fear, that technological systems may overlook. To support meaningful use of technology, some physiotherapists highlighted the importance of understanding how technological outputs are generated. In some clinics, designated physiotherapists with greater technical expertise supported colleagues in the use of the clinic’s equipment. AI was viewed as holding potential for improving planning and tailoring of training. The physiotherapists nevertheless remained sceptical as to whether AI algorithms could substitute the interpretive and intuitive aspects of professional judgement required to understand individual patients.

*”Not without our own intuition…I think someone needs to interpret everything. Someone needs to make sense of what the AI says… the strength… the jumping ability… we’re talking about that intuition that’s disappearing…”* (Focus group 6 - participant 21).

#### Utilizing increased technology-based information about the patient

The physiotherapists anticipated wider use of technology but recognised a need for support in handling the growing amount of patient information it may generate. They worried that when information becomes too extensive to review, it risks limiting the translation of meaningful findings into clinical decision making. For example, some physiotherapists noted that questionnaire data could already be difficult to review within the constraints of a busy clinic. This led them to emphasise the need for systems that help manage and prioritise information.

*”The software must have come a long way. I’ve spent hours analysing these kinds of videos myself… It takes countless hours per video before you can even get any meaningful data*,* so AI would need to step in if this is going to be clinically useful.”* (Focus group 5 - participant 13).

Although they acknowledged limited knowledge about AI, the physiotherapists saw potential for such methods to extract key patient information and support more efficient clinical reasoning.

*“We ask the patients a lot*,* when maybe we shouldn´t focus on everything… there I’d like better identification…bring in AI…Who is this person coming to me? What should I focus on? Some kind of technological pre-screening would be great…whether this is a classic movement-fearful patient or someone with complete overconfidence.”* (Focus group 6 - participant 19).

### Extending rehabilitation beyond the clinical walls

The physiotherapists described how technology could support the extension of rehabilitation beyond the clinical setting, both by addressing sport-like demands and enabling remote monitoring and movement feedback. Two sub-themes described this extension: a) Mimicking sport-like demands requires adaptations in clinical practice and b) Developing clinical solutions for monitoring and feedback.

#### Mimicking sport-like demands requires adaptations in clinical practice

The physiotherapists saw potential in using technology to better mimic the demands of sport in rehabilitation. To support these efforts, some physiotherapists asked patients to share training videos and list key sport-specific actions to better understand sport demands and tailor rehabilitation. The physiotherapists described light reaction training systems, such as BlazePods, and smartphone applications as promising tools for incorporating reaction and decision-making elements into rehabilitation. They suggested that such technologies could reduce the need for continuous cueing, allowing them to focus more on observing movement quality and performance. Achieving this potential, however, required adaptations in clinical practice as well as shifts in mindset. These included new ways of structuring rehabilitation, creativity, sport-specific knowledge, and time for individualisation, which were not always feasible within time-pressured clinical settings. The physiotherapists also described difficulties recreating sport-like environments due to limited space, a lack of unpredictability, and non-authentic surfaces in gym facilities. Although VR was not used, the physiotherapists perceived potential for such systems to support more sport-like environments and safer progression of training demands, particularly if integrated with movement analysis to strengthen RTS assessments. The physiotherapists described adapting both clinical practice and available technologies in attempts to better reflect sport-specific demands, emphasising that exercises should remain simple and easy for patients to understand and perform.

*“To replicate the situations that patients will be exposed to*,* but without the risk elements. That is something that could be developed further.”* (Focus group 6 - participant 18) /…/ *“It’s a bit unclear sometimes how to approach it [VR]. It’s really about having a sport-specific mindset early on in rehab*,* in most of what you do. But with those [VR glasses]*,* you could definitely develop it and make it more fun. But yeah*,* it’s also a matter of cost and time to really get into it. And will it be used a lot? It’s hard to know*,* but I do think there’s potential there.”* (Focus group 1 - participant 3).

#### Developing clinical solutions for monitoring and feedback

The physiotherapists described a vision to extend supervision and movement feedback beyond clinical sessions to support motor learning. In the absence of standardised methods for remote monitoring, the physiotherapists described a development of pragmatic solutions grounded in clinical needs. Attempts to monitor movement via smartphones or webcams were described as challenging due to poor image quality, limited camera angles, and unstable internet connections. The physiotherapists therefore expressed a desire for approaches that better meet clinical demands.

*“…the patients get a training programme*,* and not all training sessions are done with me. Then it would be of interest to see how they actually perform the exercises when I’m not around. What does it look like when they add more weight and load?”* (Focus group 3 - participant 8).

Alongside monitoring, the physiotherapists emphasised the importance of delivering feedback that is visually accessible and motivating for patients. Digital graphs illustrating patient progress were described as useful for supporting patients who doubted their progress and for managing expectations among those who overestimated their readiness to RTS. Motivation was also reported to increase when progress became measurable and, potentially comparable to peers, introducing an element of competition. The physiotherapists described pragmatic uses of simple technologies to support patient motivation and reflect progress in response to clinical needs.

*“I remind them of the process…This is how you did squats with this weight a month ago…It’s such a long time and it’s such a big project*,* and they’re living in it all the time. A good method is having them record themselves*,* which I help them with. Every other week*,* and then they make a kind of collage*,* they’re so good at that*,* and they can use it as a motivational thing.“* (Focus group 1 - participant 4).

## Discussion

The findings show that the physiotherapists perceived technology as potentially useful for extending rehabilitation beyond the clinical setting, provided that it adds clear clinical value. A key finding was the lack of consensus among physiotherapists regarding how to define movement quality and identify meaningful sensorimotor outcomes, influencing how technological outputs were interpreted and applied. Accordingly, the physiotherapists emphasised the need for sensorimotor outputs that are clinically meaningful, interpretable, feasible, and context-sensitive.

A central implication of our findings is the physiotherapists desire for sensorimotor outcomes to be clinically validated against relevant patient outcomes to be meaningfully implemented in clinical settings. This includes demonstrating associations with patient performance and re-injury risk. While some physiotherapists viewed movement quality as a central rehabilitation target, others questioned whether alterations in movement pattern translate into meaningful patient outcomes. The use of current clinical assessments and RTS criteria after ACL injury may not successfully reduce re-injury rates [41, 42] and these criteria have also been criticised for focusing primarily on simple performance outcomes, not incorporating sensorimotor control [43]. As a result, persistent alterations in movement patterns may be overlooked, despite being suggested to play a role in reducing re-injury risk [44]. Although structured approaches to assess movement patterns exist [45], its association with re-injury risk after ACL injury remains insufficiently established [44, 46] and more prospective studies is needed to potentially link sensorimotor outcomes to re-injury rates. Once this knowledge gap is more covered, physiotherapists may be better positioned to understand and implement sensorimotor control assessment and training in ACL injury management.

The physiotherapists in our study also emphasised the need for context-specific and situation-driven assessments that more accurately reflect sport performance. Previous research suggests that commonly used RTS tests, such as hop tasks, primarily evaluate performance in controlled conditions and may not adequately capture the sport-specific and cognitive demands inherent to real-world sport situations [47, 48]. There is, therefore, a need to develop more sport-specific approaches to assessment and rehabilitation, and technology may play an important role in supporting this process.

Although emerging technologies such as wearable sensors, pressure insoles, and video-based analyses show promise for capturing movement in sport-specific contexts, most research has focused on validation against laboratory gold standards rather than clinical applicability. Only a small proportion of studies (4%) in a review investigating portable movement analysis systems in ACL rehabilitation evaluated clinical validity or integration into care pathways [22]. This gap between research and clinical applicability may explain why physiotherapists in our study expressed doubts about the value of emerging technologies. Previous research consistently emphasises that a clear perceived clinical benefit is essential for technology adoption and implementation [49, 50]. Together, these findings suggest that without clinically meaningful outcomes, technological advancements are unlikely to translate into routine clinical practice.

The physiotherapists in our study also emphasised the need to balance technological objectivity with professional judgement to achieve meaningful evaluations. A qualitative systematic review supports this view, highlighting the importance of clinician involvement when using feedback-based technologies to achieve therapeutic benefit [51]. Consistent with this, research in stroke rehabilitation suggests that technology-based balance assessments may confirm physiotherapists’ clinical reasoning and support more precise evaluation of progress, while their impact on treatment decisions remains uncertain [52]. Together, these findings highlight the need for further research on how clinical expertise can be incorporated into the development and use of emerging technology-based feedback solutions in rehabilitation, and under which circumstances clinician involvement is more or less critical.

Previous reviews report low readiness for implementing wearable technologies in ACL rehabilitation [27, 32]. Our findings further indicate that some physiotherapists experienced uncertainty regarding the practical use of technological devices, which limits their ability to interpret outputs and integrate them into clinical reasoning. These challenges can be understood through the lens of eHealth literacy, which encompasses the ability to engage with digital technologies in effective, safe, and meaningful ways [53, 54]. While often discussed in relation to patients, emerging research highlights the importance of clinicians’ digital competencies for successful implementation [55]. Our findings, together with previous research [29, 31] suggest that hands-on engagement with technology may foster a more clinically grounded understanding of both its potential and limitations. Our results suggest a dynamic process in which the physiotherapists continuously shape and adapt technological approaches to align with clinical needs. Similar patterns have been observed in video-based rehabilitation, where clinicians undergo a reorientation of practice through trial and error [56]. Consequently, strengthening technological competence among clinicians may not only improve confidence in using digital tools but may also support the development and implementation of technologies aligned with clinical needs.

Taken together, these findings highlight key priorities for future research and clinical development. A primary focus should be the establishment of clinically meaningful and validated sensorimotor outcomes that can guide technological development. In parallel, future research should explore how movement quality is conceptualised in physiotherapy practice and how clinical expertise can be systematically integrated into technological assessment approaches. Educational initiatives may support physiotherapists in critically evaluating and applying technological outputs in clinical practice. Finally, co-creation among physiotherapists, researchers, and technology developers may help ensure that emerging technologies are aligned with rehabilitation needs. Given the limited familiarity with available technologies among the physiotherapists in this study, collaborative initiatives such as workshops and end-user evaluations could facilitate physiotherapists’ engagement with emerging technologies and support the identification of clinically relevant applications for ACL assessment and rehabilitation.

### Methodological considerations

A strength of the study was the diversity in participants’ demographics, technological familiarity, and clinical perspectives, which supported the credibility and relevance of the findings across similar rehabilitation contexts. The study was conducted in Sweden, a relatively small and highly digitalised country, which may contribute to a healthcare workforce that is more accustomed to adopting and using new technologies than is the case in some other countries. Transferability to healthcare systems in other countries should therefore be considered in light of differences in digital infrastructure, available resources, and healthcare organisation. Differences in legislation, regulatory oversight, and the maturity of e-health implementation may also affect the relevance of these findings across European and other international contexts. The analysis was informed by a reflexive thematic approach [35]. Throughout the analysis process, attention was given to the influence of the researchers’ backgrounds as physiotherapists with research experience and familiarity with rehabilitation technologies. During coding and theme development, deliberate efforts were made to remain close to participants’ clinical language and to avoid premature use of research-derived concepts. At the same time, knowledge of rehabilitation technologies was used analytically to identify aspects of novelty, while continuously checking these interpretations against the data to ensure they reflected participants’ perspectives rather than researchers’ preconceptions. Throughout the analytic process, reflexive attention was applied to identify instances where prior assumptions might have influenced the analysis and to consider alternative interpretations grounded in the data. The facilitator’s prior professional familiarity with three physiotherapists was not considered to have influenced how views were expressed, given the non-sensitive nature of the discussions. To minimise potential power imbalances, both the focus group discussions and the analysis were grounded in the physiotherapists’ own clinical language and reasoning. As sensorimotor control is not a term routinely used in clinical physiotherapy practice, it was briefly introduced to establish a shared point of reference rather than as terminology the physiotherapists were expected to adopt. Although the term itself was not explicitly used by participants, their descriptions reflected key components of sensorimotor control, including motor output through movement analysis, sensory input related to visual and surface information, and central processing evident in reflections on fear of re-injury and its influence on movement execution. Although the brief introduction to rehabilitation technologies may have influenced the physiotherapists’ reflections, it facilitated more informed engagement with emerging technologies addressed in current research and clinical practice. While larger focus groups are often recommended [33], the smaller group sizes facilitated inclusive participation and in-depth discussion within the digital format. One group included only two physiotherapists, which may have limited perspective diversity, although consensus was not an aim, and the pair still generated rich insights.

## Conclusion

The physiotherapists expressed a generally positive attitude towards technology to support the assessment and rehabilitation of sensorimotor control following ACL injury. However, implementation appears to be influenced by uncertainty regarding which sensorimotor outcomes are clinically meaningful and how technological outputs can support clinical decision-making. Establishing clinically validated sensorimotor outcomes, potentially associated with re-injury risk and functional performance, should therefore be a priority for both research and clinical practice. Future efforts should focus on strengthening physiotherapists’ engagement with emerging technologies and fostering collaboration between physiotherapists, researchers, and technology developers to ensure that technological innovation is aligned with clinical needs.

## Supplementary Information

Supplementary Material 1.

## Data Availability

The datasets generated and analysed during the current study are not publicly available to protect the participants’ confidentiality (in accordance with The General Data Protection Regulation, European Union Regulation) but are available from the corresponding author upon reasonable request.

## Abbreviations

ACL: Anterior cruciate ligament
RTS: Return to sport
VR: Virtual reality
AI: Artificial intelligence
COREQ: Consolidated criteria for reporting qualitative research
