# Supplementary material for: “It’s a black box”: Physiotherapists’ perspectives on sensorimotor control and the use of technology in this context to improve rehabilitation following ACL injury
Source: BMC Musculoskelet Disord. 2026 Sep 23;27:840. doi: 10.1186/s12891-026-10484-x (PMC13644143; doi:10.1186/s12891-026-10484-x)
Supplement: Supplementary file 1 [file 12891_2026_10484_MOESM1_ESM.docx]

S-Fig. 1. Interview guide used in the focus group discussions

S-Table 1. The table presents different technologies that were directly mentioned in the focus groups, their current use and/or visions for their potential applications. The condensed statements presented in the table were directly derived from the focus group sessions while retaining participants' terminology where possible. This table should not be seen as a comprehensive overview of available or desirable technologies in general but provides illustrative examples of what the physiotherapists referred to in their discussions, however without specific prompts for such statements (out of the scope of the current study).

|  | **Technology mentioned** | **Currently used for** | **Visions for potential application** |
| --- | --- | --- | --- |
| **Evaluation** | Wearable movement sensors; e.g., IMUs (gyros and accelerometers),  Smartphone friendly movement lab and applications | Measure jump height | Remote movement and asymmetry assessment in everyday activities, compare joint angles in clinical and functional tasks, hip control, detailed assessment of sport-specific tasks, detect re-injury risk factors, running step length, identification of factors contributing to setbacks and overload  Movement screening for re-injury prevention |
|  | 3D motion capture system and force plates | Jump test analysis | Joint-specific force generation in jump tests, loading in running, sway, proprioception |
|  | Pressure sensors | Jump test analysis | Assessment of side-to-side loading asymmetries during squatting, contact, assessment of contact time in side-hop |
|  | Video recordings | Provide feedback for patients on their movement pattern during functional tasks, recall bias for patients on their progression, track rehabilitation progress over time, slow-motion jump analysis, sport-specific movement analysis | Motion capture analysis, remote estimation of loading during rehabilitation exercise |
|  | Isokinetic dynamometer, handheld dynamometer, Nordic board | Muscle strength testing |  |
|  | Muscle-lab system | Jump flight time, muscle strength testing, limb loading symmetry |  |
|  | Electromyography |  | Fear and anxiety of movement |
|  | Virtual reality glasses |  | Sport-specific testing in limited gym facilities, simulation of sport-specific environments, structured progression of sport-specific skills |

| **Training/**  **rehabilitation** | Smartphone applications | Reaction training |  |
| --- | --- | --- | --- |
|  | Laser pointer | Knee control training |  |
|  | Blaze pods | Training reactive movements to sensory stimuli |  |
|  | Neuromuscular electrical stimulation | Muscle stimulation |  |
| **Communication and support** | Artificial intelligence methods | Creating presentations for patients and colleagues | Movement data analysis, development of training programs tailored to patient characteristics, automated detection of movement patterns and abnormalities, monitoring movement changes over time, guide clinical decision-making based on patient characteristics and movement data |
|  | Telehealth consultation | Visual assessment of walking and rehabilitation exercise | Consultations regarding post-exercise symptoms, increase exercise adherence and guide optimal training load |
|  | Electronic questionnaire data collection | Digital collection of questionnaire information |  |
|  | Projector and camera with wireless headphones, e.g., GoPro |  | Video-based exercise instruction and supervision |
|  | Digital training programme digital information films | Digital instructions of rehabilitation exercises (text, pictures and videos) | National patient information videos |
